# Non-Stick Length of Polymer–Polymer Interfaces under Small-Amplitude Oscillatory Shear Measurement

**DOI:** 10.3390/polym16010077

**Published:** 2023-12-26

**Authors:** Yasuya Nakayama

**Affiliations:** Department of Chemical Engineering, Kyushu University, Nishi-ku, Fukuoka 819-0395, Japan; nakayama@chem-eng.kyushu-u.ac.jp

**Keywords:** immiscible polymers, polymer–polymer interface, interfacial rheology, linear viscoelasticity

## Abstract

Interfaces in soft materials often exhibit deviation from non-slip/stick response and play a determining role in the rheological response of the overall system. We discuss detection techniques for the excess interface rheology using small-amplitude oscillatory shear (SAOS) measurements. A stacked bilayer of different polymers is sheared parallel to the interface and the dynamic shear response is measured. Deviation of the bilayer shear modulus from the superposition of the shear moduli of the component layers is analysed. Furthermore, we introduce a frequency-dependent non-stick length based on the bilayer SAOS response to characterize the excess interface rheology. We observe an approximate stick response in the interface in bilayers composed of the chemically same monomer as well as an apparent slip in the interface between immiscible polymers. The results suggest that the proposed non-stick length in SAOS is capable of detecting the apparent interfacial slip. The non-stick length in SAOS is readily applicable to other complex interfaces of different soft materials and offers a convenient tool to characterize the excess interface rheology.

## 1. Introduction

Interfaces in soft materials often deviate from a simple stick rheological behavior at an infinitely small thickness of a simple interface, and are thus called complex interfaces. Complex interfaces are common in industrial materials, foods, and biological systems. An interface between polymers is one typical example of a complex interface.

Issues of interest regarding the interface rheology of polymeric flows include the drag friction of a droplet [1,2], the coalescence kinetics of droplets in immiscible blends and emulsions [3], the viscosity reduction in polymer blends [4,5] and emulsions [6] by interfacial slip, multilayer melt flow in coextrusion processing [7], adhesion properties in relation to the effect of melt rheological processing [8], and the interface formation in multi-material 3D printing [9]. Understanding these phenomena requires the characterization of not only the bulk rheology of the components but also of the interface rheology.

Several methods for characterizing the rheology of complex interfaces have been developed. For air–liquid and liquid–liquid interfaces, several types of surface shear rheometries have been applied [10,11]. A microrheology technique has also been exploited for air–liquid and liquid–liquid interfaces [12,13,14]. Atomic force microscopy has been used to characterize the local rheological properties of the surface of polymeric materials [15,16]. In these techniques, probes should directly contact the interface. Therefore, their application is limited to systems that allow the probes to be immersed in one phase, such as air or a low-viscosity liquid.

The rheology of stacked multilayer polymer melts has been used to study the influence of polymer–polymer interfaces. Steady shear flow and dynamic shear measurements parallel to the interface have been used to characterize the interfacial slip [17,18,19,20,21,22]. In contrast to the immiscible interface, the shear response of the multilayer film with compatibilized interfaces indicates solid-like behavior of the interphase [23,24]. The pressure drop in the multilayer coextrusion process was measured and compared with that of the non-slip condition at the interface to detect the interfacial slip under steady shear flow [25]. Deviation from the stick condition has also been reported in stress relaxation measurements [26]. The interdiffusion of miscible polymers has been characterized by dynamic shear measurements [27], and elongation measurements have been used to measure interfacial tension [28]. Furthermore, an increased transient elongational viscosity and strain hardening are observed in multilayer film even when the constituent polymers exhibited no strain-hardening behavior, indicating extra stress contribution of the interphase [29,30,31]. The Mooney method that was originally developed to characterize the wall slip under steady flow was applied to the interfacial slip [32]. Using this modified Mooney method, the two power-law regimes for the slip velocity as a function of shear stress were observed.

Compatibilizers are often used to suppress slippage at interfaces and improve the processability and physical properties of multilayer products [33,34]. The compatibilization mechanism and the rheology of the compatibilizing interface of different types of compatibilizers have been studied. Interphase nonlinear relaxation of multilayer film compatibilized in situ reaction in coextrusion has been reported [35]. Viscosity increases and strain hardening of multilayer films have been reported at interfaces compatibilized with branched copolymer [36]. Coarse-grained molecular dynamics simulations have been applied to study the mechanism and rheology of compatibilized interphase by diblock copolymer [37], Janus nanorod [38], dumbbell-shaped Janus polymer particle [39], and clay particle [40]. The rheology of multilayer polymer melts is a convenient tool for studying an immersed interface. However, the quantification of the influence of the interface is still a challenging task.

The apparent slip or possibly more complex excess response of the interface is supposed to originate from the existence of a finite-volume interphase that is mechanically different from the bulk materials. In the previous work [22], the dynamic shear modulus of the interphase was estimated by explicitly assuming the thickness of the interphase predicted by the Flory–Huggins theory [41,42,43], which is at most several nanometers. However, it is not trivial whether it is appropriate to assume that the thickness of the interphase, whose non-equilibrium response is different from the bulk layers, is equal to the equilibrium thickness. Experimental measurements of local thermal conductivity indicate that the interphase between immiscible polymers spans several micrometers [44,45] which is much larger than the equilibrium interface thickness estimated by the Flory—Huggins and Helfand—Tagami theories [46,47]. This experimental fact indicates an issue concerning the physical property of an interface. The region where the interface-specific response, not only thermal conductivity but also other ones including mechanical response which is different from the bulk responses, is larger than the region of the equilibrium thickness which is characterized by inhomogeneity of composition at equilibrium state. The interface-specific rheological response can also be involved in a region larger than the equilibrium thickness.

The difficulty stems from the fact that the thickness of the rheological interphase is generally unknown and hard to determine. Nevertheless, since the evaluation of the excess rheological contribution of the interphase is an important issue, a robust technique independent of the interphase thickness is desired. In this article, we discuss detection techniques for the excess interface rheology under small-amplitude oscillatory shear (SAOS) measurements and propose a frequency-dependent non-stick length as a technique which does not require an explicit interphase thickness.

## 2. Experimental Section

### 2.1. Materials

The polymers used in this study are listed in Table 1. Two polystyrenes (PS) of different molecular weight distributions were obtained from the PS Japan Corporation (Tokyo, Japan): PSJ-Polystyrene™680 (PS680) and 685 (PS685). Two different linear low-density polyethylenes (LLDPE) were obtained from the Japan Polyethylene Corporation (Tokyo, Japan): NOVATEC™-LL UJ960 and UF230. A high-density polyethylene (HDPE) (NOVATEC™-HD(HY540)) was also obtained from Japan Polyethylene Corporation. The molecular weight distributions of these polymers were estimated using gel permeation chromatography. The polymers were obtained in pellet form.

### 2.2. Sample Preparation

The polymer pellets were dried for at least 24 h at 100 ${}^{\circ }$C in a vacuum oven prior to use. Each polymer was compression-moulded at a temperature of approximately 60 ${}^{\circ }$C above its melting point or glass-transition point in a hot press to form a plaque and then cooled to room temperature. The thickness of each polymer plaque was kept at approximately 1 mm using a spacer frame made of stainless steel so that the plaque would be suitable for the rheological measurements described below. Each polymer melt was pressed between two sheets of polytetrafluoroethylene (Naflon™PTFE from NICHIAS Corporation, Tokyo, Japan).

### 2.3. Rheological Measurements

Polyethylene (PE) and polystyrene (PS) are immiscible, so they are expected to form a loosely sticking interface with some slippery rheology. We focused on pairs of linear low-density polyethylenes (LLDPE) and polystyrenes as immiscible pairs. In contrast, linear low-density polyethylenes and high-density polyethylenes (HDPE) are based on a chemically same monomer but they are not completely mixed due to having different chain architectures and molecular weight distributions. Therefore, they are expected to form a rheologically sticky interface. In order to study the response of a stick interface, we also tested pairs of linear low-density polyethylenes and high-density polyethylenes.

For both the pristine polymers and the bilayers, the dynamic shear moduli were measured at different temperatures under a nitrogen atmosphere using a rotational rheometer (Rheosol-G1000, UBM, Kyoto, Japan) in parallel-plate geometry with a plate diameter of 25 mm. The oscillatory shear measurement was performed over the frequency range of 0.1–62.8 rad/s. The strain amplitude was set at approximately 0.2 or lower, falling within the linear response regime in all of the pristine polymers and bilayers, as identified through preliminary amplitude sweep measurements. The small-amplitude oscillatory shear measurements for a polymer were performed at least three times.

**Figure 1:**
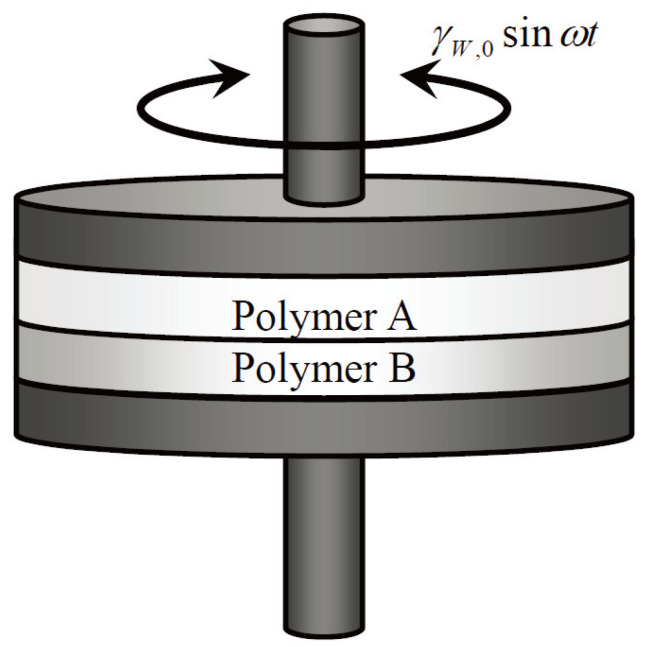
Experimental setup of dynamic shear measurement of a bilayer sample. Oscillatory rotational shear deformation is applied parallel to the interface formed between different polymers A and B.

The bilayer samples were prepared in the sample chamber of the rheometer (Figure 1). We loaded two different polymer plaques in the sample chamber and raised the chamber temperature to a given measurement temperature to melt the polymers. At the measurement temperature, the gap between the parallel plates was compressed to approximately 2 mm. To equilibrate the polymer–polymer interface, we held each bilayer at its measurement temperature for 180 s. Subsequently, small-amplitude oscillatory shear measurements were performed.

## 3. Non-Stick Length in Small-Amplitude Oscillatory Shear Response of an Interface

**Figure 2:**
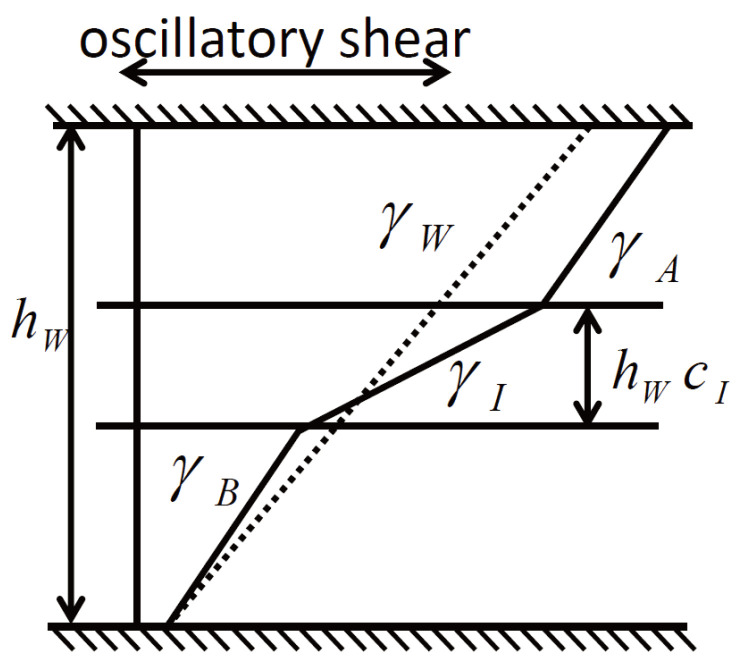
Schematic of the displacement across an interfacial layer. The apparent shear deformation of the whole system, $\gamma_{W}$, can be decomposed into the deformation of bulk responses of both polymers, $\gamma_{A}$ and $\gamma_{B}$, and the possible excess deformation of the interfacial layer, $\gamma_{I}$, which is different from those of the adjacent bulk layers. We put the thickness of the interfacial layer as $h_{W}c_{I}$.

In a previous work [22], we discussed how to determine the dynamic shear modulus of an interfacial layer from the dynamic shear response of the bilayer of two different polymers. The rheological interfacial layer is defined as the region around the interface where the response to the shear deformation is different from that of neighboring bulk layers (Figure 2). A difficulty in the evaluation of the modulus of the interfacial layer is that it requires a determination of the thickness of the interfacial layer. Estimating the interface thickness is rather difficult in general, especially in non-equilibrium situations, and directly affects the accuracy of the interfacial shear modulus. It would be desirable to have a different approach for characterizing the excess rheological contribution of the interface, one without using the interface thickness. In this direction, we propose a method to evaluate the non-stick length of the interface under small-amplitude dynamic shear measurements.

We here derive a frequency-dependent non-stick length for the excess rheological contribution of the interface through small-amplitude oscillatory shear measurements of a bilayer sample. If the materials are in the linear response regime, the response of each layer is described as

(1) $$\begin{array}{cc} \tau & =G_{\alpha }^{*}\left(\omega \right)\gamma_{\alpha },\;\;\left(\alpha =W,A,B,I\right) \end{array}$$

where τ is the shear stress, $G_{\alpha }^{*}\left(\omega \right)$ is the dynamic shear modulus of the αth layer at a frequency ω, $\gamma_{\alpha }=\gamma_{\alpha ,0}e^{i\omega t}$ is the oscillatory strain of the αth layer with an amplitude $\gamma_{\alpha ,0}$ as depicted in Figure 2, and the subscripts W,A,B, and *I* indicate the whole bilayer, material A, material B, and the interface layer, respectively.

Note that we explicitly consider the finite interface layer and its dynamic shear modulus. The shear stress τ is constant throughout the bilayer system. The stress on material A is transmitted to material B through the interface between them; therefore, the deformation of each layer is continuous throughout the sample. Namely, the total displacement of the whole bilayer system $\Delta x_{W}$ is equal to the sum of the displacement in each layer $\Delta x_{\alpha }$ as $\Delta x_{W}=\sum_{\alpha =A,B,I}\Delta x_{\alpha }$. The displacement in the αth layer is expressed with its thickness fraction $c_{\alpha }$ and $\gamma_{\alpha }$ as $\Delta x_{\alpha }=h_{W}c_{\alpha }\gamma_{\alpha }$ with $h_{W}$ the thickness of the bilayer sample. Since $\sum_{\alpha =A,B,I}c_{\alpha }=1=c_{W}$ holds by definition, $\Delta x_{W}=h_{W}\gamma_{W}$. This leads to the following relation between the strains of different layers:

(2) $$\begin{array}{cc} \gamma_{W} & =c_{A}\gamma_{A}+c_{B}\gamma_{B}+c_{I}\gamma_{I}. \end{array}$$

Combining Equations (1) and (2), the following relation is obtained:

(3) $$\begin{array}{cc} \frac{1}{G_{W}^{*}} & =\frac{c_{A}}{G_{A}^{*}}+\frac{c_{B}}{G_{B}^{*}}+\frac{c_{I}}{G_{I}^{*}}. \end{array}$$

This Equation (3) is derived from the displacement continuity and defines the dynamic shear modulus of the interphase layer $G_{I}^{*}$ and the (non-equilibrium) thickness fraction of the interphase layer $c_{I}$, where the interphase thickness $h_{I}=h_{W}c_{I}$ is defined as the scale of the region that exhibits different response than the bulk monolayers, and is not necessarily equal to the equilibrium interface thickness by the Flory–Huggins and Helfand–Tagami theories [46,47]. To determine the interphase shear modulus $G_{I}^{*}$ from Equation (3), thickness fractions, $c_{A},c_{B}$, and $c_{I}$ are required. However, the determination of $c_{I}$ is practically rather difficult. Moreover, $c_{A}$ and $c_{B}$ are dependent on $c_{I}$ due to the equation $\sum_{\alpha =A,B,I}c_{\alpha }=1$. Therefore, it is difficult to directly evaluate the interphase shear response from Equation (3) since Equation (3) has three unknown variables, $c_{I},G_{I}^{*}$, and $c_{A}$ (or equivalently $c_{B}$). In past studies [18,19,48,49,50], the interphase thickness is neglected as $c_{I}\to 0$ and $c_{A}+c_{B}=1$ to define the quantities called slip index which is the relative displacement in the interphase to that in the whole bilayer, and the energy factor which is kind a slip index based solely on the loss modulus. However, the validity of this approximation is not obvious if we consider the interphase shear response at the infinitely thin region. Therefore, it would be desirable to have another approach to characterize the excess interphase response that does not require the interphase thickness fraction.

We here explain how macroscopic stick response is described in the model of Equation (3). Since the interface is physically characterized by a region with a composition different from those of the bulk layers, $c_{I}>0$ holds in general, not $c_{I}=0$. When the interface exhibits macroscopically stick response, the interphase shear modulus $G_{I}^{*}$ should be similar to the shear moduli of the bulk layers. As an example, suppose the interphase shear modulus is given by the harmonic mean of the shear moduli of the bulk layers as $1/G_{I}^{*}=\left(1/2\right)\left(1/G_{A}^{*}+1/G_{B}^{*}\right)$, by assuming that both A- and B-components equally contribute to the interphase shear modulus without any interaction effects between them, the bilayer shear modulus becomes $1/G_{W}^{*}=\left(c_{A}+c_{I}/2\right)/G_{A}^{*}+\left(c_{B}+c_{I}/2\right)/G_{B}^{*}$ where the weight $c_{\alpha }+c_{I}/2$ (α=A,B) is equal to the volume fraction of the αth material supposing that the distribution in the interphase is split evenly between two component phases.

If the interface term in Equation (3) is omitted, the interface contribution to the overall response, if it exists, is accounted for by the effective thickness fractions, $c_{A}^{\mathrm{eff}}$ and $c_{B}^{\mathrm{eff}}$, as

(4) $$\begin{array}{cc} \frac{1}{G_{W}^{*}} & =\frac{c_{A}^{\mathrm{eff}}}{G_{A}^{*}}+\frac{c_{B}^{\mathrm{eff}}}{G_{B}^{*}}, \end{array}$$

where $G_{W}^{*}$, $G_{A}^{*}$ and $G_{B}^{*}$ are determined by experimental measurement. A complex shear modulus consists of two independent components: $G^{*}=G^{\prime }+iG^{\prime \prime }=\left|G^{*}\right|\exp\left(i\delta \right)$. Here, we assume that $c_{A}^{\mathrm{eff}}$ and $c_{B}^{\mathrm{eff}}$ are real numbers to proceed to the following analysis. Then, Equation (4) becomes the following simultaneous linear equation for $c_{A}^{\mathrm{eff}}$ and $c_{B}^{\mathrm{eff}}$,

$$\begin{array}{cc} \frac{1}{\left|G_{W}^{*}\right|}\left(\begin{array}{c} \cos\delta_{W}\\ \sin\delta_{W} \end{array}\right)\; & =\left(\begin{array}{cc} \frac{\cos\delta_{A}}{\left|G_{A}^{*}\right|}\; & \frac{\cos\delta_{B}}{\left|G_{B}^{*}\right|}\\ \frac{\sin\delta_{A}}{\left|G_{A}^{*}\right|}\; & \frac{\sin\delta_{B}}{\left|G_{B}^{*}\right|} \end{array}\right)\left(\begin{array}{c} c_{A}^{\mathrm{eff}}\\ c_{B}^{\mathrm{eff}} \end{array}\right)=\left(\begin{array}{cc} \cos\delta_{A}\; & \cos\delta_{B}\\ \sin\delta_{A}\; & \sin\delta_{B} \end{array}\right)\left(\begin{array}{cc} \frac{1}{\left|G_{A}^{*}\right|}\; & 0\\ 0\; & \frac{1}{\left|G_{B}^{*}\right|} \end{array}\right)\left(\begin{array}{c} c_{A}^{\mathrm{eff}}\\ c_{B}^{\mathrm{eff}} \end{array}\right). \end{array}$$

This assumption means that the interphase shear modulus $G_{I}^{*}$ is represented by a harmonic mean of $G_{A}^{*}$ and $G_{B}^{*}$

(5) $$\begin{array}{cc} \frac{c_{I}}{G_{I}^{*}} & =\frac{c_{A}^{\mathrm{eff}}-c_{A}}{G_{A}^{*}}+\frac{c_{B}^{\mathrm{eff}}-c_{B}}{G_{B}^{*}}, \end{array}$$

which equation indicates that $c_{A}^{\mathrm{eff}}$ and $c_{B}^{\mathrm{eff}}$ are related to the weights that relate the monolayer shear moduli $G_{A}^{*}$ and $G_{B}^{*}$ to the interphase shear modulus $G_{I}^{*}$ rather than the physical thickness fractions.

By solving Equation (4), $c_{A}^{\mathrm{eff}}$ and $c_{B}^{\mathrm{eff}}$ are obtained as

(6) $$\begin{array}{cc} \left(\begin{array}{c} c_{A}^{\mathrm{eff}}\\ c_{B}^{\mathrm{eff}} \end{array}\right)\; & =\frac{1}{\left|G_{W}^{*}\right|\sin\left(\delta_{B}-\delta_{A}\right)}\left(\begin{array}{c} \left|G_{A}^{*}\right|\sin\left(\delta_{B}-\delta_{W}\right)\\ \left|G_{B}^{*}\right|\sin\left(\delta_{W}-\delta_{A}\right) \end{array}\right). \end{array}$$

Technically, when the phase lags of both polymers $\delta_{A}$ and $\delta_{B}$ coincide, the coefficient matrix in Equation (4) is singular so that Equation (4) cannot be solved. This fact simply means that even if the substances of each monolayer are different, if the dynamical shear response of each monolayer is the same, the effective thickness fraction cannot be inferred by the dynamic shear response. Around this singular point, $\delta_{A}\approx \delta_{B}$, due to the ill condition of the coefficient matrix, the solution tends to diverge. In order to circumvent this technical difficulty and obtain reliable values of $c_{A}^{\mathrm{eff}}$ and $c_{B}^{\mathrm{eff}}$, a minimal phase lag difference for $\left|\delta_{B}-\delta_{A}\right|$ should be introduced when using Equation (6). The prefactor in Equation (6), $1/\sin\left(\delta_{B}-\delta_{A}\right)$, is approximated as $1/\left(\delta_{B}-\delta_{A}\right)$ when the phase lag difference is small enough. For a phase lag difference larger than a several degree, $1/\left(\delta_{B}-\delta_{A}\right)$ varies slowly enough. Therefore, we arbitrarily choose a small threshold value for the phase lag difference as we restrict our analysis to the data of $\left|\delta_{A}-\delta_{B}\right|>3^{\circ }$.

We note that $c_{A}^{\mathrm{eff}}$ and $c_{B}^{\mathrm{eff}}$ in Equation (4) can be defined as complex numbers, However, the physical interpretation of such complex $c_{A}^{\mathrm{eff}}$ and $c_{B}^{\mathrm{eff}}$ is not clear. Furthermore, two complex numbers cannot be uniquely determined by Equation (4) only; this requires an additional condition on $c_{A}^{\mathrm{eff}}$ and $c_{B}^{\mathrm{eff}}$ which should be related to the physical interpretation of the complex $c_{A}^{\mathrm{eff}}$ and $c_{B}^{\mathrm{eff}}$. Such extension of Equation (4) is beyond the scope of this article.

Based on $c_{A}^{\mathrm{eff}}$ and $c_{B}^{\mathrm{eff}}$, the non-stick length of the interface is defined as

(7) $$\begin{array}{cc} h_{s}\left(\omega \right) & =h_{W}\left(c_{A}^{\mathrm{eff}}\left(\omega \right)+c_{B}^{\mathrm{eff}}\left(\omega \right)-1\right). \end{array}$$

If no interface contribution exists, the relation is expected to be $c_{A}^{\mathrm{eff}}+c_{B}^{\mathrm{eff}}=1$ leading to $h_{s}=0$. Otherwise, a nonzero $h_{s}$ indicates an interface contribution to the bilayer shear modulus. A positive $h_{s}$ indicates a larger strain or a more viscous response in the interface layer than in the bulk layers (cf. Figure 2). In this case, $h_{s}$ is a counterpart of the slip length, $l=\Delta V_{I}/\dot{\gamma }$ with the velocity jump $\Delta V_{I}$ and the shear rate $\dot{\gamma }$, in slippage under steady shear flow [51], not small-amplitude oscillatory shear deformation. In contrast, a negative $h_{s}$ indicates a smaller strain or a more elastic response in the interface layer than in the bulk layers. In short, the different signs of $h_{s}$ reflect different qualitative contributions of the interface layer to the bilayer shear modulus.

Note that although $h_{s}$ defined in Equation (7) has a dimension of length, it is a quantity that reflects the rheological response of the interphase rather than the physical scale of the interphase. In other words, $h_{s}$ value can be comparable to $h_{W}$ when the interphase shear response is very soft or liquid-like and thus the apparent strain of the bilayer system $\gamma_{W}$ becomes larger than that in the stick interface response, and is larger than the physical interphase thickness $h_{W}c_{I}$. The essential point is that $h_{s}$ reflects the deviation of $c_{A}^{\mathrm{eff}}+c_{B}^{\mathrm{eff}}$ from 1. This fact is similar to the fact that the slip length in steady shear flow $l=\Delta V_{I}/\dot{\gamma }$ reflects the largeness of the velocity jump and does not mean the real physical length.

## 4. Results and Discussion

**Figure 3:**
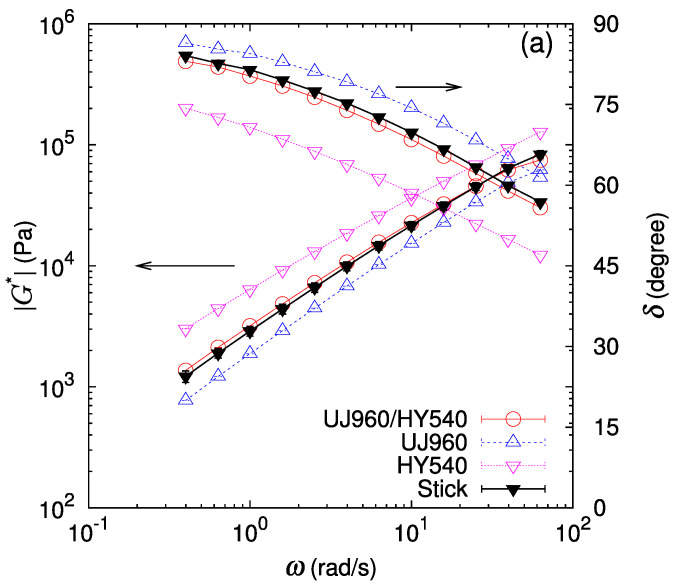

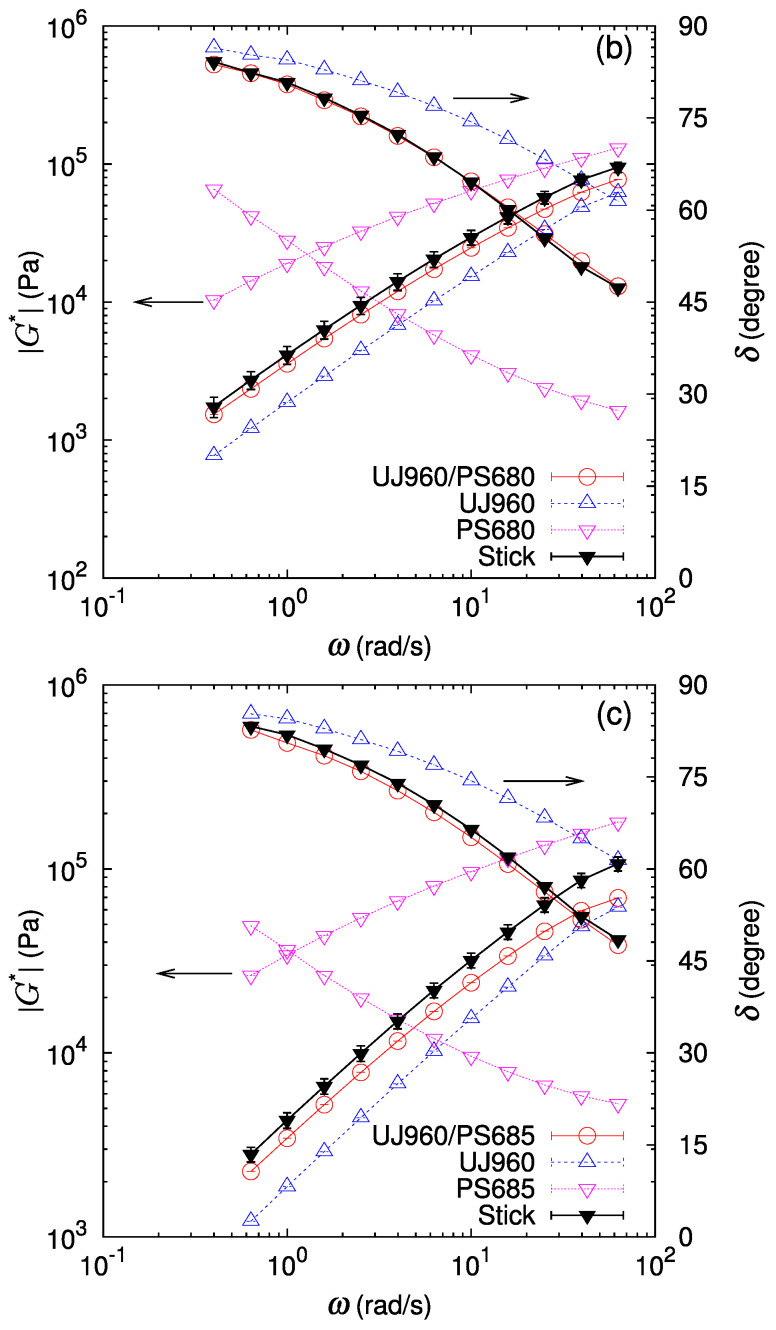
Amplitude of the complex shear modulus $\left|G^{*}\right|$ and phase lag δ at 190 ${}^{\circ }$C for bilayers of UJ960 (LLDPE) and different polymers (red open circle). For comparison, data for each polymer (blue open upper triangle and pink open lower triangle) and the prediction assuming stick interface (black filled triangle) are also drawn. (**a**) UJ960/HY540 (LLDPE/HDPE) pair, (**b**) UJ960/PS680 (LLDPE/PS) pair, and (**c**) UJ960/PS685 (LLDPE/PS) pair. Lines are guides to the eye.

**Figure 4:**
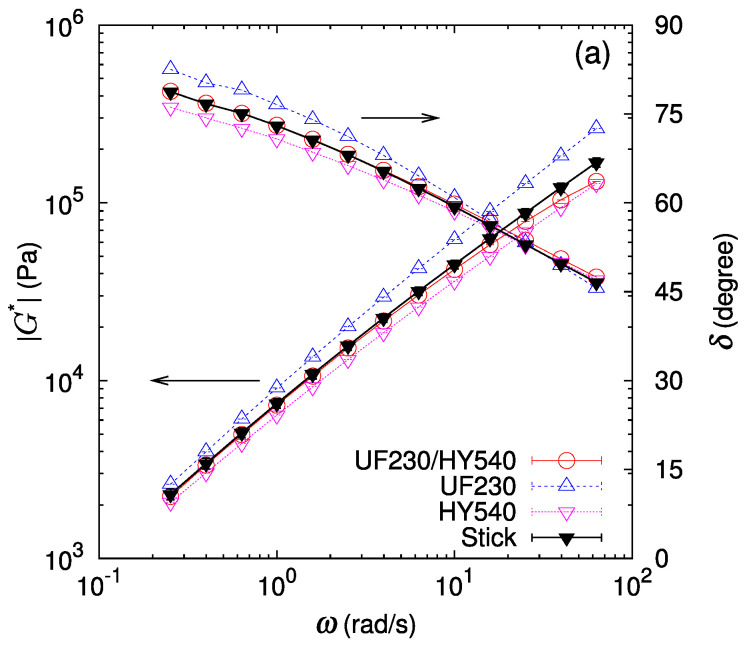

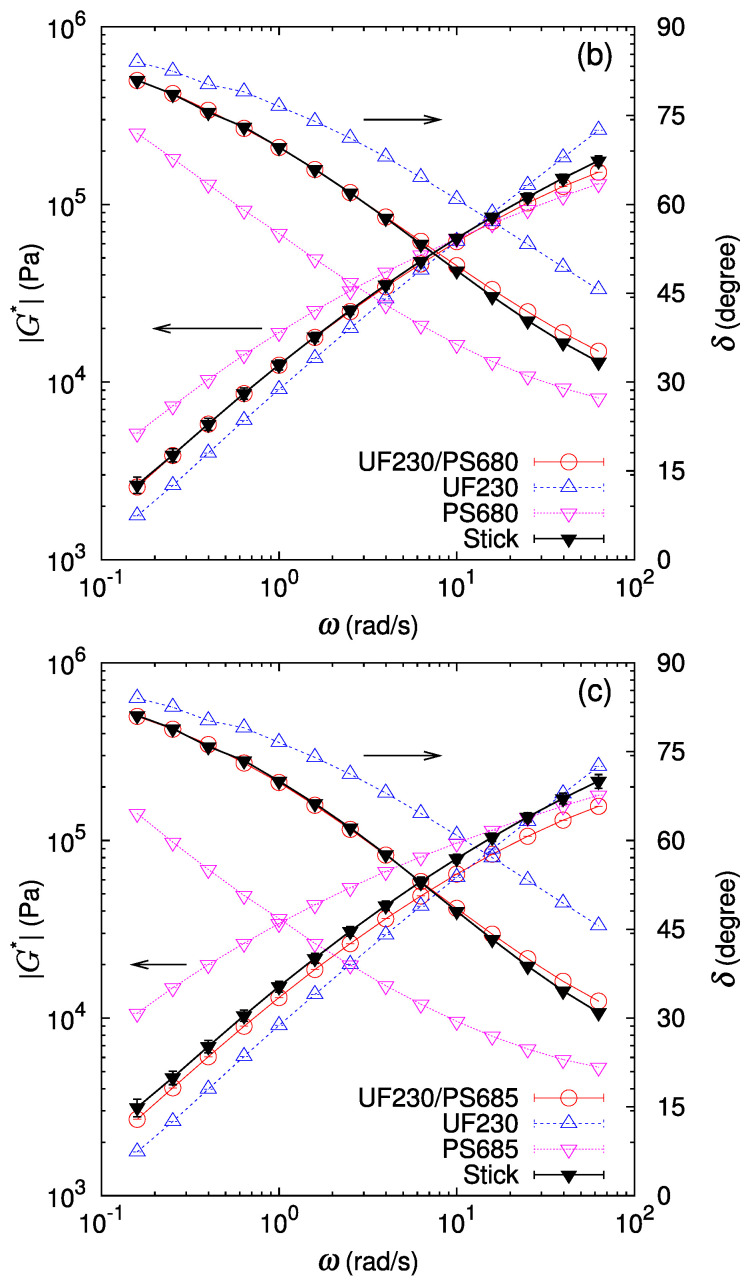
Amplitude of the complex shear modulus $\left|G^{*}\right|$ and phase lag δ at 190 ${}^{\circ }$C for bilayers of UF230 (LLDPE) and different polymers (red open circle). For comparison, data for each polymer (blue open upper triangle and pink open lower triangle) and the prediction assuming stick interface (black filled triangle) are also drawn. (**a**) UF230/HY540 (LLDPE/HDPE) pair, (**b**) UF230/PS680 (LLDPE/PS) pair, and (**c**) UF230/PS685 (LLDPE/PS) pair. Lines are guides to the eye.

The linear shear moduli of bilayer samples $G_{W}^{*}$ (red open circle) are compared with those of the monolayer samples $G_{A}^{*}$ and $G_{B}^{*}$ (blue and pink triangles) in Figure 3 and Figure 4. At first glance, we observe that the shear modulus amplitude and the phase lag of the bilayer lie between those of the component polymers in most cases. One exception is the case of the UF230/PS685 (LLDPE/PS) bilayer (Figure 4c) where we observe that at high frequencies the shear modulus amplitude of the bilayer $\left|G_{W}^{*}\right|$ is lower than those of both component polymers $\left|G_{A}^{*}\right|$ and $\left|G_{B}^{*}\right|$, indicating a substantial apparent slip at the interface.

For a more detailed analysis, a prediction for the bilayer shear modulus assuming the stick condition at the interface with vanishing thickness is calculated according to Equation (3) by setting $c_{I}=0$. The thickness fraction of each layer is determined by measuring thicknesses of both component layers in the bilayer sample set in the parallel plate in melt state at the measurement temperature. This stick prediction $G_{\mathrm{stick}}^{*}$ (black filled triangle) is also drawn in Figure 3 and Figure 4. In most cases, the phase lag of the bilayer samples almost coincides with that of the stick prediction. In contrast, for the shear modulus amplitude of the bilayer samples, while the stick prediction works well for some cases (UJ960/HY540 (LLDPE/HDPE), UF230/HY540 (LLDPE/HDPE), and UF230/PS680 (LLDPE/PS)), a lower shear modulus than predicted by the stick assumption is noticeable for the other cases (UJ960/PS680 (LLDPE/PS), UJ960/PS685 (LLDPE/PS), UF230/PS685 (LLDPE/PS)). Furthermore, the deviation from the stick prediction shows a frequency dependence.

**Figure 5:**
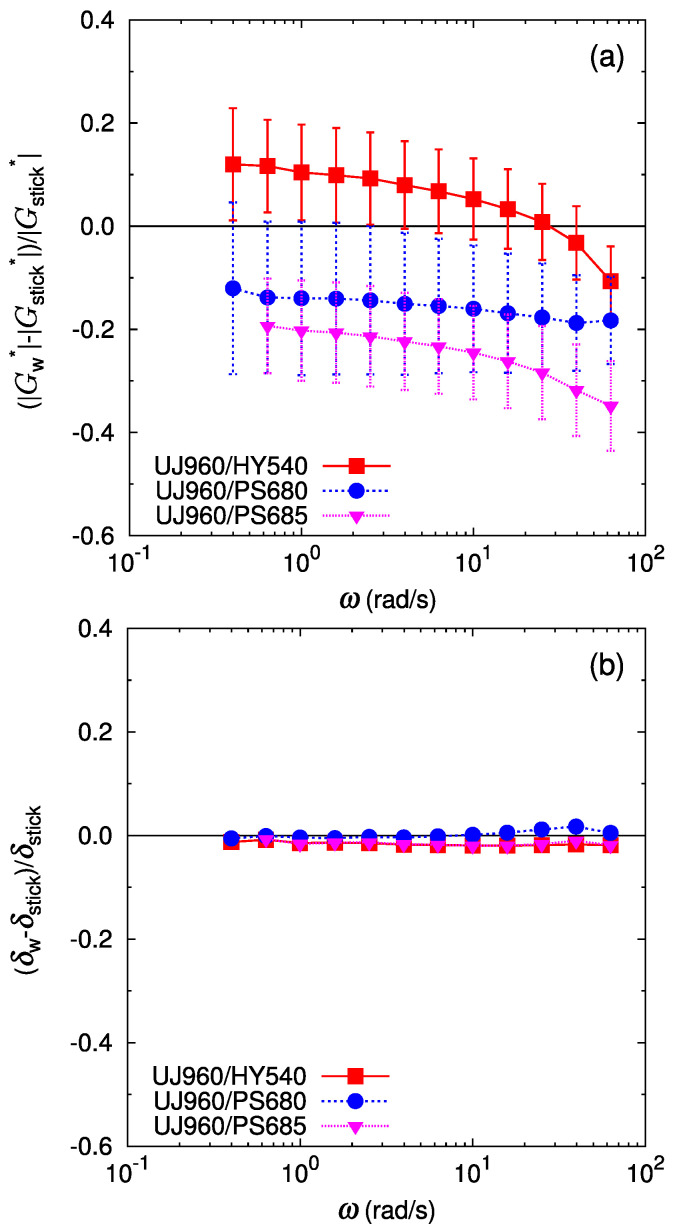
Deviation of the bilayer shear modulus from the prediction by the stick interface for the bilayers of UJ960 (LLDPE) and different polymers, HY540 (HDPE) (red filled square), PS680 (PS) (blue filled circle), and PS685 (PS) (pink filled triangle): (**a**) for the amplitude of the complex shear modulus, (**b**) for the phase lag of the complex shear modulus. Lines are guides to the eye.

**Figure 6:**
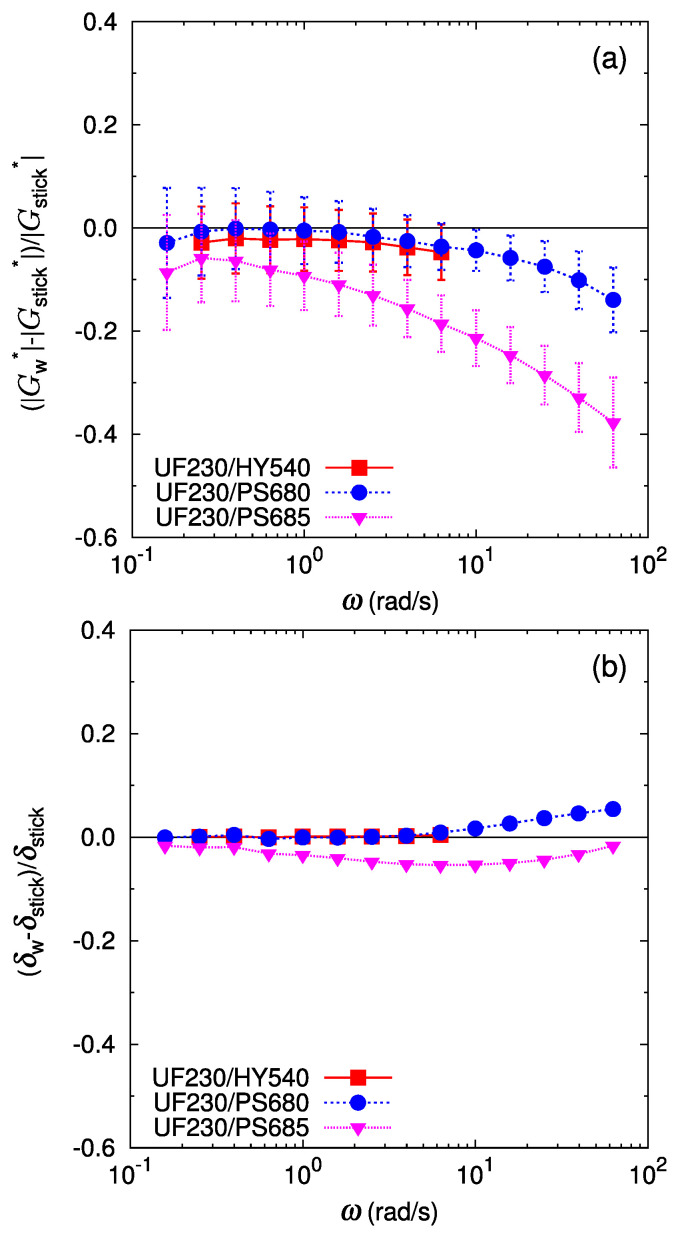
Deviation of the bilayer shear modulus from the prediction by the stick interface for the bilayers of UF230 (LLDPE) and different polymers, HY540 (HDPE) (red filled square), PS680 (PS) (blue filled circle), and PS685 (PS) (pink filled triangle): (**a**) for the amplitude of the complex shear modulus, (**b**) for the phase lag of the complex shear modulus. Lines are guides to the eye.

The relative deviation of the bilayer shear modulus from the stick prediction is shown in Figure 5 and Figure 6. As mentioned above, the deviation of the phase lag $\delta_{W}$ from the stick prediction $\delta_{\mathrm{stick}}$ is not substantial for all the cases (Figure 5b and Figure 6b). In Figure 5b, the deviation of the phase lag from the stick response for UJ960/PS680 (LLDPE/PS) shows a slight increasing trend at ω>20 rad/s. Qualitatively, the larger delta of the bilayer shear response than that of the stick response might indicate a more solid-like response in the bilayer system. However, quantitatively, this deviation is less than a few percent, and is difficult to regard as substantial. For the LLDPE/HDPE case of the UF230/HY540 pair (Figure 6a), no significant deviation of the bilayer shear modulus amplitude $\left|G_{W}^{*}\right|$ from the stick prediction $\left|G_{\mathrm{stick}}^{*}\right|$ is detected (difference is only within error bars), as expected because the polymers are chemically the same and therefore have no special interactions at the interface. For another LLDPE/HDPE case of the UJ960/HY540 pair (Figure 5a), the average deviation of $\left|G_{W}^{*}\right|$ from $\left|G_{\mathrm{stick}}\right|$ varies around zero; the average relative deviation of $\left|G_{W}^{*}\right|$ is positive at low frequencies, then decreases with frequency, but the deviation still falls within error bars. Except at the highest frequency in Figure 5a, no softening by the interface is observed, i.e., $\left|G_{W}^{*}\right|\approx \left|G_{\mathrm{stick}}^{*}\right|$ for UJ960/HY540 (LLDPE/HDPE) pair.

In contrast, for immiscible pairs of LLDPE/PS, the deviation of $\left|G_{W}^{*}\right|$ from the stick prediction is significant and depends on the pair. For the UJ960(LLDPE)/PS pairs in Figure 5a, the average relative deviation of $\left|G_{W}^{*}\right|$ is more than 10% lower than $\left|G_{\mathrm{stick}}^{*}\right|$. Furthermore, this negative deviation increases with an increase in the frequency. These facts clearly indicate that an apparent slip occurs at the interface between UJ960 (LLDPE) and PS due to the reduced shear modulus amplitude of the interfacial layer.

In the cases containing another LLDPE of UF230 shown in Figure 6, basically relative deviation of the bilayer shear modulus of UF230(LLDPE)/PS shows similar trends as in UJ960(LLDPE)/PS. However, at low frequencies, $\left|G_{W}^{*}\right|$ of UF230(LLDPE)/PS is much closer to that of the stick prediction than of UJ960(LLDPE)/PS. Especially, $\left|G_{W}^{*}\right|$ of UF230/PS680 (LLDPE/PS) almost coincides with the stick prediction at low frequencies, indicating no apparent slip in slow deformation.

In summary, the results suggest that in small-amplitude oscillatory shear measurement, the stick assumption approximately holds for an LLDPE/HDPE interface which consists of chemically same monomers, and an apparent slip occurs in immiscible LLDPE/PS interfaces due to the lower shear modulus amplitude of the interfacial layer. In addition, the apparent slip is more enhanced at high frequencies.

**Figure 7:**
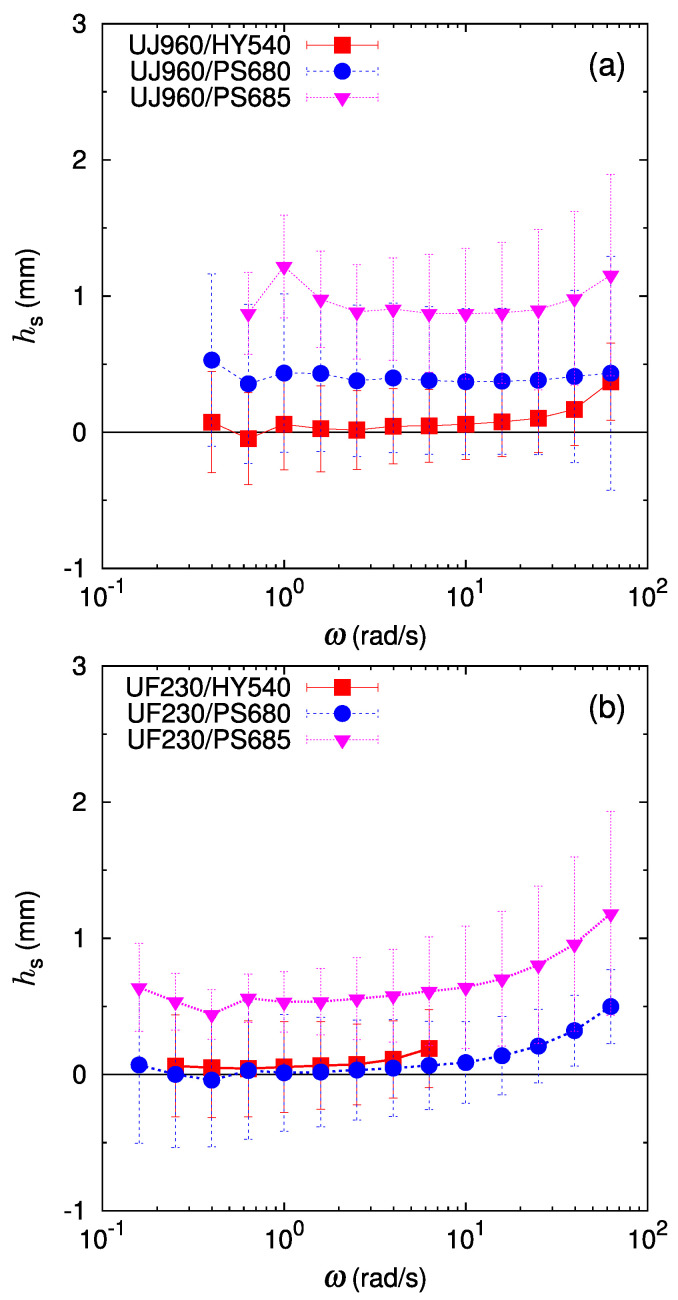
Frequency-dependent interface non-stick length: (**a**) the interface between UJ960 (LLDPE) and different polymers (HY540 (HDPE) (red filled square), PS680 (PS) (blue filled circle), and PS685 (PS) (pink filled triangle), and (**b**) the interface between UF230 (LLDPE) and different polymers. Lines are guides to the eye.

Next, we discuss the frequency-dependent non-stick length, $h_{s}$, defined in Equation (7). A difficulty in analyses using stick prediction comes from the fact that the estimation of the stick prediction requires the thickness fraction in the bilayer sample in melt state, yet an accurate determination of the thicknesses in the stacked bilayer sample is difficult in the melt state. In contrast, the non-stick length $h_{s}$ in Equation (7) can be evaluated without knowing the thickness fractions, and still measures the excess rheological contribution of the interface. Figure 7 shows the non-stick length $h_{s}$ as a function of frequency for the cases in Figure 5 and Figure 6. For both LLDPE/HDPE cases of the UJ960/HY540 pair (Figure 7a) and the UF230/HY540 pair (Figure 7b), we observe $h_{s}\approx 0$, which clearly indicates a stick response at the LLDPE/HDPE interface. In LLDPE/PS pairs, a positive $h_{s}$ is observed in the UJ960/PS680 pair, the UJ960/PS685 pair (Figure 7a), and the UF230/PS685 pair (Figure 7b), suggesting a substantial apparent slip at these interfaces. For the UF230/PS680 pair (Figure 7b), $h_{s}$ almost vanishes at low frequencies, and increases to be positive at high frequencies. These observations of $h_{s}$ are basically consistent with the comparison with the stick prediction. From these results, the frequency-dependent non-stick length $h_{s}$ turns out to be useful to detect the degree of apparent slip at the interface in SAOS measurement of a bilayer sample, without using the thickness of each layer.

## 5. Conclusions

We discussed the detection of the excess rheological contribution of the interface in a stacked bilayer sample in small-amplitude oscillatory shear (SAOS) measurement. Two approaches were tested for quantification of the apparent interfacial slip. One approach is the comparison between the bilayer shear modulus and the shear modulus predicted by assuming a stick condition at the interface with vanishing thickness. As another approach, we developed a frequency-dependent non-stick length in SAOS measurement. Both approaches worked well to characterize the stick response at LLDPE/HDPE interfaces which consist of chemically same monomers as well as the apparent slip at LLDPE/PS interfaces which are interfaces of immiscible polymers. The apparent slip in LLDPE/PS interface turns out to be frequency-dependent and becomes stronger as the frequency increases. The degree of the apparent slip by the non-stick length is basically consistent with the results of direct comparison with the stick prediction. One difficulty of the analysis using stick prediction is that the result depends on the accuracy of the measurement of the thickness of each layer in melt state. In contrast, the non-stick length can be evaluated without the thickness of each layer, which is advantageous in the detection of the excess rheology of the interface in different situations.

The frequency-dependent non-stick length can be readily applied to other systems having complex interfaces with internal structures. Basically, the SAOS response has been a convenient and useful probe to study the internal relaxation processes in many kinds of soft materials. The non-stick length based on the SAOS response of a stacked layer sample offers a new way to quantify the excess rheological response of various complex interfaces. Therefore, the method proposed in this paper can have a wide range of applications in complex interfaces, including copolymer compatibilized interfaces, colloid/nanoparticle-absorbed interfaces, and biological interfaces.

## Figures and Tables

**Table 1:** Molecular weights of the polymer samples.

Polymer	$M_{w}$ (kg/mol)	$M_{w}/M_{n}$
polystyrene (PS)		
PS680	187	2.5
PS685	247	2.4
linear low-density polyethylene (LLDPE)		
UJ960	78	3.2
UF230	265	3.7
high-density polyethylene (HDPE)		
HY540	301	7.6

## Data Availability

Data are contained within the article.

## References

[1] Boussinesq J. (1913). Existence of a superficial viscosity in the thin transition layer separating one liquid from another contiguous fluid. CR Hehbd. Seanc. Acad. Sci..

[2] Happel J., Brenner H. (1973). Low Reynolds Number Hydrodynamics: With Special Applications to Particulate Media (Mechanics of Fluids and Transport Processes).

[3] Yu W., Zhou C. (2008). The effect of interfacial viscosity on the droplet dynamics under flow field. J. Polym. Sci. Part B Polym. Phys..

[4] Tanaka Y., Sako T., Hiraoka T., Yamaguchi M., Yamaguchi M. (2020). Effect of morphology on shear viscosity for binary blends of polycarbonate and polystyrene. J. Appl. Polym. Sci..

[5] Moonprasith N., Nasri M.S., Saari R.A., Phulkerd P., Yamaguchi M. (2021). Viscosity decrease by interfacial slippage between immiscible polymers. Polym. Eng. Sci..

[6] Das S., Bhattacharjee A., Chakraborty S. (2018). Influence of interfacial slip on the suspension rheology of a dilute emulsion of surfactant-laden deformable drops in linear flows. Phys. Fluids.

[7] Wilson H.J. (2006). Instabilities and constitutive modelling. Philos. Trans. R. Soc. A Math. Phys. Eng. Sci..

[8] Zhang J., Lodge T.P., Macosko C.W. (2006). Interfacial slip reduces polymer–polymer adhesion during coextrusion. J. Rheol..

[9] Lopes L., Silva A., Carneiro O. (2018). Multi-material 3D printing: The relevance of materials affinity on the boundary interface performance. Addit. Manuf..

[10] Fuller G.G., Vermant J. (2012). Complex Fluid–Fluid Interfaces: Rheology and Structure. Annu. Rev. Chem. Biomol. Eng..

[11] Pelipenko J., Kristl J., Rošic R., Baumgartner S., Kocbek P. (2012). Interfacial rheology: An overview of measuring techniques and its role in dispersions and electrospinning. Acta Pharm..

[12] Prasad V., Koehler S.A., Weeks E.R. (2006). Two-Particle Microrheology of Quasi-2D Viscous Systems. Phys. Rev. Lett..

[13] Song Y., Dai L.L. (2010). Two-Particle Interfacial Microrheology at Polymer–Polymer Interfaces. Langmuir.

[14] Moghaddam R.K., Roy T., Natale G. (2022). Interfacial microrheology: Characteristics of homogeneous and heterogeneous interfaces. Rheol. Acta.

[15] Tanaka K., Takahara A., Kajiyama T. (2000). Rheological Analysis of Surface Relaxation Process of Monodisperse Polystyrene Films. Macromolecules.

[16] Wang D., Fujinami S., Nakajima K., Nishi T. (2010). True Surface Topography and Nanomechanical Mapping Measurements on Block Copolymers with Atomic Force Microscopy. Macromolecules.

[17] Zhao R., Macosko C.W. (2002). Slip at polymer–polymer interfaces: Rheological measurements on coextruded multilayers. J. Rheol..

[18] Lam Y.C., Jiang L., Yue C.Y., Tam K.C., Li L., Hu X. (2003). Interfacial slip between polymer melts studied by confocal microscopy and rheological measurements. J. Rheol..

[19] Jiang L., Lam Y.C., Zhang J. (2005). Rheological properties and interfacial slip of a multilayer structure under dynamic shear. J. Polym. Sci. B Polym. Phys..

[20] Lee P.C., Park H.E., Morse D.C., Macosko C.W. (2009). Polymer–polymer interfacial slip in multilayered films. J. Rheol..

[21] Park H.E., Lee P.C., Macosko C.W. (2010). Polymer–polymer interfacial slip by direct visualization and by stress reduction. J. Rheol..

[22] Nakayama Y., Kataoka K., Kajiwara T. (2013). Dynamic shear responses of polymer–polymer interfaces. Nihon Reoroji Gakkaishi (J. Soc. Rheol. Jpn.).

[23] Beuguel Q., Guinault A., Léger L., Restagno F., Sollogoub C., Miquelard-Garnier G. (2019). Nanorheology with a conventional rheometer: Probing the interfacial properties in compatibilized multinanolayer polymer films. ACS Macro Lett..

[24] Beuguel Q., Guinault A., Chinesta F., Sollogoub C., Miquelard-Garnier G. (2020). Modeling of the rheological properties of multinanolayer films in the presence of compatibilized interphase. J. Rheol..

[25] Zhao R., Macosko C.W. (2007). Polymer–polymer mutual diffusion via rheology of coextruded multilayers. AIChE J..

[26] Silva J., Maia J.a.M., Huang R., Meltzer D., Cox M., Andrade R. (2012). Interfacial rheology of coextruded elastomeric and amorphous glass thermoplastic polyurethanes. Rheol. Acta.

[27] Qiu H., Bousmina M. (1999). New technique allowing the quantification of diffusion at polymer/polymer interfaces using rheological analysis: Theoretical and experimental results. J. Rheol..

[28] Levitt L., Macosko C.W., Schweizer T., Meissner J. (1997). Extensional rheometry of polymer multilayers: A sensitive probe of interfaces. J. Rheol..

[29] Jordan A.M., Lee B., Kim K., Ludtke E., Lhost O., Jaffer S.A., Bates F.S., Macosko C.W. (2019). Rheology of polymer multilayers: Slip in shear, hardening in extension. J. Rheol..

[30] Gholami F., Pakzad L., Behzadfar E. (2020). Morphological, interfacial and rheological properties in multilayer polymers: A review. Polymer.

[31] Dmochowska A., Peixinho J., Sollogoub C., Miquelard-Garnier G. (2023). Extensional Viscosity of Immiscible Polymer Multi-Nanolayer Films: Signature of the Interphase. Macromolecules.

[32] Komuro R., Sukumaran S.K., Sugimoto M., Koyama K. (2014). Slip at the interface between immiscible polymer melts I: Method to measure slip. Rheol. Acta.

[33] Lu B., Zhang H., Maazouz A., Lamnawar K. (2021). Interfacial phenomena in multi-micro-/nanolayered polymer coextrusion: A review of fundamental and engineering aspects. Polymers.

[34] Dziadowiec D., Matykiewicz D., Szostak M., Andrzejewski J. (2023). Overview of the Cast Polyolefin Film Extrusion Technology for Multi-Layer Packaging Applications. Materials.

[35] Zhang H., Lamnawar K., Maazouz A. (2018). Understanding of Transient Rheology in Step Shear and Its Implication to Explore Nonlinear Relaxation Dynamics of Interphase in Compatible Polymer Multi-microlayered Systems. Ind. Eng. Chem. Res..

[36] Lu B., Bondon A., Touil I., Zhang H., Alcouffe P., Pruvost S., Liu C., Maazouz A., Lamnawar K. (2020). Role of the Macromolecular Architecture of Copolymers at Layer–Layer Interfaces of Multilayered Polymer Films: A Combined Morphological and Rheological Investigation. Ind. Eng. Chem. Res..

[37] Li Y., Guo H. (2023). Nonequilibrium Behaviors of Entangled Diblock Copolymers at the Entangled Polymer–Polymer Interface under Steady Shear Flow. J. Phys. Chem. B.

[38] Paiva F.L., Secchi A.R., Calado V., Maia J., Khani S. (2020). Slip and momentum transfer mechanisms mediated by Janus rods at polymer interfaces. Soft Matter.

[39] Qiao H., Zheng B., Zhong G., Li Z., Cardinaels R., Moldenaers P., Lamnawar K., Maazouz A., Liu C., Zhang H. (2023). Understanding the rheology of polymer–polymer interfaces covered with Janus nanoparticles: Polymer blends versus particle sandwiched multilayers. Macromolecules.

[40] Saha S., Xu D., Gersappe D. (2021). Effect of compatibilizers on the structure and dynamics at polymer blend interfaces. Tribol. Lett..

[41] Mark J.E. (2006). Physical Properties of Polymers Handbook.

[42] Brandrup J., Immergut E.H., Grulke E.A. (1999). Polymer Handbook.

[43] Young R.J., Lovell P.A. (2011). Introduction to Polymers.

[44] Kotera M., Urushihara Y., Izumo D., Nishino T. (2012). Interfacial structure of poly-*α*-olefin laminate by using scanning thermal microscope. Thermochim. Acta.

[45] Kotera M., Urushihara Y., Izumo D., Nishino T. (2012). Interfacial structure of all-polyethylene laminate using scanning thermal microscope and nano-Raman spectroscope. Polymer.

[46] Helfand E., Tagami Y. (1971). Theory of the interface between immiscible polymers. J. Polym. Sci. B Polym. Lett..

[47] Helfand E., Tagami Y. (1972). Theory of the Interface between Immiscible Polymers. II. J. Chem. Phys..

[48] Jiang L., Lam Y.C., Yue C.Y., Tam K.C., Li L., Hu X. (2003). Energy model of the interfacial slip of polymer blends under steady shear. J. Appl. Polym. Sci..

[49] Lam Y.C., Yue C.Y., Yang Y.X., Tam K.C., Hu X. (2003). Interfacial properties of polycarbonate/liquid-crystal polymer and polystyrene/high-impact polystyrene polymer pairs under shear deformation. J. Appl. Polym. Sci..

[50] Lam Y.C., Jiang L., Li L., Yue C.Y., Tam K.C., Hu X. (2004). Interfacial slip at the thermotropic liquid-crystalline polymer/poly (ethylene naphthalate) interface. J. Polym. Sci. B Polym. Phys..

[51] de Gennes P.G. (1979). Viscometric flows of tangled polymers. Comptes Rendus Hebd. Des Seances Acad. Des Sci. Ser. B.

